# The SPECTRAL Perfusion Arm Clamping dAtaset (SPECTRALPACA) for video-rate functional imaging of the skin

**DOI:** 10.1038/s41597-024-03307-y

**Published:** 2024-05-25

**Authors:** Leonardo Ayala, Diana Mindroc-Filimon, Maike Rees, Marco Hübner, Jan Sellner, Silvia Seidlitz, Minu Tizabi, Sebastian Wirkert, Alexander Seitel, Lena Maier-Hein

**Affiliations:** 1 German Cancer Research Center (DKFZ), Division of Intelligent Medical Systems, Heidelberg, Germany; 2 Medical Faculty, Heidelberg University, Heidelberg, Germany; 3 Faculty of Mathematics and Computer Science, Heidelberg University, Heidelberg, Germany; 4 Helmholtz Information and Data Science School for Health, Karlsruhe/Heidelberg, Germany; 5 National Center for Tumor Diseases (NCT) Heidelberg, a partnership between DKFZ and Heidelberg University Hospital, Heidelberg, Germany

**Keywords:** Medical research, Computational biology and bioinformatics, Scientific data

## Abstract

Spectral imaging has the potential to become a key technique in interventional medicine as it unveils much richer optical information compared to conventional RBG (red, green, and blue)-based imaging. Thus allowing for high-resolution functional tissue analysis in real time. Its higher information density particularly shows promise for the development of powerful perfusion monitoring methods for clinical use. However, even though *in vivo* validation of such methods is crucial for their clinical translation, the biomedical field suffers from a lack of publicly available datasets for this purpose. Closing this gap, we generated the SPECTRAL Perfusion Arm Clamping dAtaset (SPECTRALPACA). It comprises ten spectral videos (∼20 Hz, approx. 20,000 frames each) systematically recorded of the hands of ten healthy human participants in different functional states. We paired each spectral video with concisely tracked regions of interest, and corresponding diffuse reflectance measurements recorded with a spectrometer. Providing the first openly accessible *in human* spectral video dataset for perfusion monitoring, our work facilitates the development and validation of new functional imaging methods.

## Background & Summary

The enhanced perception of tissues and structures that are invisible to the naked eye, conventionally by RGB (red, green, and blue)-based imaging, has become indispensable for various types of interventional clinical procedures. As these imaging methods mimic the function of the human eye by collecting light in three broad spectral regions (red, green, and blue), they inherently limit practitioners to the morphological analysis of tissue. However, the ability to functionally assess tissue in real time is crucial in a number of medical interventions, such as partial kidney resection (nephrectomy)^1^, anastomosis^2,3^, and wound monitoring^4,5^.

Addressing this clinical need, spectral imaging (SI)^2^ techniques have evolved as a promising alternative. In contrast to RGB imaging, SI is capable of collecting light in many more and narrower regions of the optical spectrum. This property, in combination with tissue-specific spectral signatures, caused by the unique interaction of different molecules with light, enables SI to encode optical information of much higher density than traditional imaging techniques, ultimately allowing for the development of methods that offer functional tissue assessment in real time. Over the last decade, many approaches to functional tissue monitoring *in vivo*, notably of perfusion and oxygenation, have emerged, using both gray-scale and spectral imaging. These approaches can be classified into two main categories: a) model-based approaches^2,4–10^, and b) approaches that leverage machine learning-based tools^1,11–14^. For the remainder of the manuscript, we will use the term perfusion to refer to properties related to the assessment of tissue perfusion such as oxygenation and blood volume fraction.

**Fig. 1:**
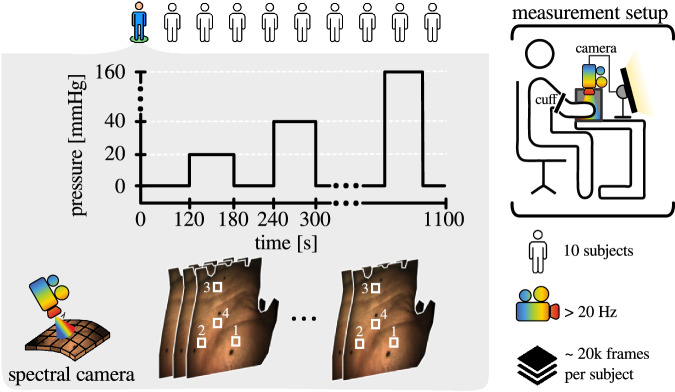
The SPECTRALPACA dataset comprises video-rate spectral hand measurements of ten participants at eight different pressure levels. Each video sequence comprises approximately 20,000 frames that were recorded over a period of approximately 18 minutes. The pressure applied to the upper right arm of each participant was increased between 20 mmHg and 160 mmHg at regular intervals of one minute, interspersed with resting intervals of approximately one minute. In addition, spectrometer measurements were recorded at the beginning of each experiment (fully perfused hand, 0 mmHg) and at maximum pressure level (160 mmHg). The white squares numerated from 1 to 4 correspond to exemplary tracked ROI that form part of the SPECTRALPACA dataset.

The majority of these methods aiming to monitor changes in perfusion *in humans* are validated in arm-clamping experiments^4,6,15–17^. The goal of such experiments is to detect changes in the oxygenation of the hand while clamping the upper arm with a pressure cuff. Even though this validation method enjoys common use in the scientific community and can be perceived as a gold standard, to date, no openly accessible dataset for this purpose exists. Instead, competing institutions currently have to acquire their own, essentially duplicate data, which renders fair method comparison unfeasible. Furthermore, it depletes resources and stalls progress in the field, ultimately hampering the clinical translation of new methods. In addition, most openly accessible spectral imaging datasets are limited to the domain of remote sensing^18,19^, botany^20^, and material science^21^. The only few openly accessible spectral imaging datasets in the medical field^22–24^ are limited to spectrometer recordings, single image recordings (no videos), and are focused on tissue discrimination rather than tissue function (e.g. oxygenation). Even though prior work on the use of spectral cameras for the analysis of skin exists^4,25,26^, their data is not openly accessible. To address such issues, we introduce the first openly accessible spectral video-rate dataset: SPECTRAL Perfusion Arm Clamping dAtaset (SPECTRALPACA)^27^ (Fig. 1). Our work presents the first openly accessible human spectral video-rate dataset covering varying functional states, where each video spans over 18 minutes and comprises approximately 20,000 spectral frames on average. To aid the validation of functional imaging methods, we have concisely tracked Regions of Interest (ROIs) on the surface of the right palm of ten healthy volunteers. Each ROI was carefully selected to avoid potential undesired effects such as specular highlights, undersaturated regions, and palmar creases. In addition, we provide diffuse reflectance spectrometer recordings for each ROI at two different pressure states. As such, this carefully crafted dataset opens the door for improved validation both in terms of quality and quantity, as well as clinical translation of functional imaging methods.

## Methods

The data described in this manuscript were carefully recorded in an *in human* study involving ten healthy volunteers. The following sections describe how the spectral data were recorded, how the ROI were annotated, and how the data were processed. All experiments involving humans were performed in accordance with the Declaration of Helsinki and all protocols were approved by the ethics commission of Heidelberg University (DE/EKBW03, study reference number: S-530/2020). The study is registered with the German Clinical Trials Register (DRKS00023246). Patient consent was obtained from all participants of this study. Participants consented to sharing the data recorded in the context of this study, provided that identification of the participants based on these data is not possible.

### Spectral data collection

**Fig. 2:**
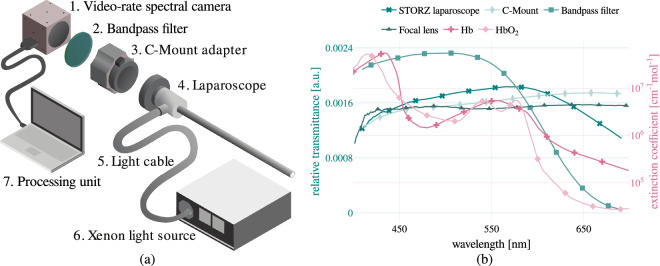
Experimental hardware (left) and transmission profiles of optical components used for data recording (right).

**Fig. 3:**
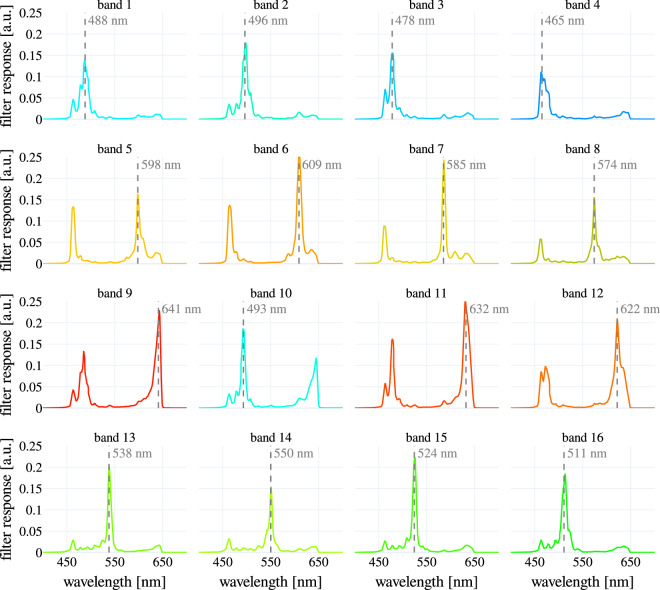
Multispectral camera (filter) responses for model MQ022HG-IM-SM4x4-VIS. Some bands, such as 5 to 12, show two distinctive peaks in the spectral response, which are referred to as “second order” peaks.

The video-rate spectral imaging data were acquired by recording images of the right palm of ten healthy volunteers with a spectral camera (MQ022HG-IM-SM4x4-VIS, XIMEA GmbH, Münster, Germany) (Fig. 2a)). This camera records 16 spectral channels in the visible region of the optical spectrum (Fig. 3). To standardize the illumination geometry in relation to the camera line of view across all participants, a 0° laparoscope (26003, KARL STORZ SE & Co. KG, Tuttlingen, Germany) was used to couple the light source (IP20, Richard Wolf GmbH, Knittlingen, Germany) and the spectral camera (Fig. 2a)). A continuous spectral video of each subject’s right palm was recorded while the pressure on the right arm was increased from 0 mmHg to 160 mmHg at regular time intervals of one minute using a manual pressure cuff (Fig. 1). The baseline measurement (0 mmHg) was conducted for approximately two minutes at the beginning of each video sequence, whereas the stages with increased pressure were recorded for one minute each to reduce stress on the subjects. To further alleviate stress on the subjects, each stage of applied pressure was followed by a resting stage (0 mmHg) of one minute. The time duration for each stage was chosen to maximize the amount of data recorded, while avoiding pain in the participants. To eliminate the effect of stray light such as sunlight or light emanating from ceiling lamps, the illumination in the room was turned off, the blinds closed, and both the spectral camera and the right hand of each subject were placed inside a black box for the entire duration of the recordings (measurement setup in Fig. 1). A standard laptop (MSI GE75 Raider 85 G, Intel i7, NVIDIA RTX 2080) running an implementation of a custom C++ software (not publicly available) based on the XIMEA Application Program Interface (XiAPI, XIMEA, Münster, Germany) was used to acquire and record the spectral images.

To complement the spectral videos, diffuse reflectance spectra were collected using a spectrometer (HR2000+, Ocean Insight, Orlando, USA), and a halogen light source (HL-2000, Ocean Insight, Orlando, USA). An optical fiber reflection probe bundle was used to illuminate and collect the reflected light from the skin (RP22, Thorlabs, Newton, United States). In this configuration, six concentric fibers were used for illumination and one single fiber at the center of the bundle was used to collect the light reflected from the skin. The reflectance probe was placed ∼5 mm away from the skin during data collection, and cleaned after each use. These measurements were performed in four different anatomical areas of the hand: the thenar region (ROI 1), the hypothenar region (ROI 2), the interdigital region (ROI 3), and the palmar region (ROI 4)^28^. A small landmark was created next to these regions using a black marker to a) aid the tracking of ROI, and b) correlate the locations where spectrometer data was recorded with the tracked ROI in the spectral videos. All four ROIs were measured before the spectral video recordings started (baseline at 0 mmHg), and again after the recordings had ended (160 mmHg). For consistency, measurements were always performed in the same order: ROI 1 → ROI 2 → ROI 3 → ROI 4.

### Region of interest annotation

Four ROIs of size 20 × 20 px, placed in the aforementioned anatomical areas of the hand, were manually annotated on the first frame of each spectral video, and subsequently automatically tracked. The precise location within each anatomical area was selected based on the requirements that a) the pixels were neither oversaturated nor undersaturated, b) the ROI locations did not overlap, c) no palmar creases were present, and d) the ROI locations corresponded to where diffuse reflectance measurements were recorded with the spectrometer (aided by created landmarks).

The tracking was conducted on RGB images reconstructed from the spectral images. This RGB reconstruction was performed by selecting bands 3, 11 and 15 (Fig. 3) from each spectral frame. Furthermore, to enable reliable tracking, the RGB images were normalized with Contrast Limited Adaptive Histogram Equalization (CLAHE)^29^. The kernel size used by CLAHE was set to 1/8 of the image height by 1/8 of its width, the number of bins was set to 256, and the clipping limit to 0.01. The reconstructed and normalized RGB images were further processed by the tracker Discriminative Correlation Filter with Channel and Spatial Reliability (CSR-DCF)^30^. Finally, each ROI was visually inspected to ensure that no visible drift from the original annotated tissue region had occurred.

### Data processing

The data contained in the SPECTRALPACA^27^ dataset are unprocessed data. However, for the analysis presented in this manuscript, the spectral videos were first calibrated with a white (*W*) and dark (*D*) reference recording taken with a highly reflective target (Spectralon®, Edmund Optics, Barrington, USA). Given an image of dimensions $N\times M\times B$, where $(N,M)$ are the spatial dimensions and *B* is the number of spectral bands, the intensity *I* of each pixel at spatial location $(i,j)$ and spectral band *k*, it was normalized according to:

1 $${({\bar{I}}_{(i,j)})}_{k}=\frac{{({I}_{(i,j)})}_{k}-{({D}_{(i,j)})}_{k}}{{({W}_{(i,j)})}_{k}-{({D}_{(i,j)})}_{k}}$$

Subsequently, an ${\ell }_{1}$-normalization across the different bands was performed to compensate for the influence of light source intensity changes due to variation in the distance of the camera to the surface of the hand:

2 $${({\widehat{I}}_{(i,j)})}_{k}=\frac{{({\bar{I}}_{(i,j)})}_{k}}{{\parallel {\bar{I}}_{(i,j)}\parallel }_{1}}\quad ;\quad {\parallel {\bar{I}}_{(i,j)}\parallel }_{1}=\mathop{\sum }\limits_{k=1}^{B}{({\bar{I}}_{(i,j)})}_{k}$$

The resulting spectra ${({\widehat{I}}_{(i,j)})}_{k}$ can be compared between different image sequences.

As part of the validation of the spectral camera, the diffuse reflectance data measured with the spectrometer $r(\lambda )$ were transformed into the spectral camera space, thus yielding the measurement ${r}_{k}$ at band *k* according to:

3 $${r}_{k}=\frac{{\int }_{{\lambda }_{min}}^{{\lambda }_{max}}{\mathcal{J}}(\lambda )\cdot J(\lambda )\cdot {f}_{k}(\lambda )\cdot r(\lambda )d\lambda }{{\int }_{{\lambda }_{min}}^{{\lambda }_{max}}{\mathcal{J}}(\lambda )\cdot J(\lambda )\cdot {f}_{k}(\lambda )d\lambda },$$

were $[{\lambda }_{min},{\lambda }_{max}]$ is the spectral range of each band *k* of the spectral camera, $I(\lambda )$ represents the optical transmission profile of the optical components of our hardware setup (Fig. 2), ${\mathcal{J}}(\lambda )$ is the relative irradiance of the light source, and ${f}_{k}(\lambda )$ characterizes the *k*^th^ optical filter response of the camera.

## Data Records

The SPECTRALPACA^27^ dataset can be downloaded from Synapse (SynID syn51625685) following the instructions provided in this link. The dataset is mainly composed of spectral videos and spectrometer diffuse reflectance data, and is organized in two levels. The first level refers to the two types of data included in the archive: spectral videos and spectrometer measurements. The spectral videos are provided as individual files for all ten subjects, which can be loaded with the sample Python code provided together with the dataset. Each spectral video is named *subject_NN.b2nd*, where *NN* represents the subject ID (see Table 1), and is stored in a lossless, highly optimized compressed format developed by BLOSC (NDArray). This facilitates fast data decompression, and loading of individual frames from each video. The spectral videos are accompanied by dark and white reference measurements that can be used to calibrate the spectral videos. In addition, the filter responses of the spectral camera and the relative irradiance of the light source used to record the spectral videos is provided as comma-separated values (CSV) and in *Feather* format for faster data loading. The spectrometer measurements for all subjects are contained in a single file, provided in both CSV and *Feather* format.

**Table 1:** Human subjects recruited for our clinical study.

subject	smoker	diabetic	age	sex
1	yes	no	22	female
2	no	no	33	female
3	no	no	24	male
4	no	no	21	male
5	no	no	29	male
6	no	no	26	male
7	no	no	32	female
8	no	no	25	female
9	no	no	35	male
10	no	no	24	female

The age of the subjects is between 21 and 35 years at the time of data recording. All subjects except for subject 1 are non-smokers, all subjects are non-diabetic, and 50% of the subjects are female.

Similarly to the spectrometer measurements, the locations of the tracked ROI (in image coordinates) for all subjects are provided in both CSV and *Feather* format. These ROI are stored in the *intermediates* directory of the dataset and separated into individual folders for each subject. Each ROI file is named *roi_N.csv*, where *N* corresponds to the ROI ID.

## Technical Validation

We performed a comprehensive analysis of the data contained in the SPECTRALPACA^27^ dataset to validate the data recording process and the data quality. The following sections describe the analysis of the imaging system used for data acquisition, with a focus on the spectral camera, and the characterization of the recorded spectral data.

### Imaging system characterization

To validate the spectral camera used to acquire the data from each subject, we recorded 24 color tiles from a color checkerboard (ColorChecker Classic, X-Rite GmbH, Planegg-Martinsried, Germany) with a spectrometer (HR2000 + , Ocean Insight, Orlando, USA), a Xenon light source (IP20, Richard Wolf GmbH, Knittlingen, Germany), and the spectral camera coupled with a 35 mm focal lens (67–716, Edmund Optics GmbH, Mainz, Germany) (Fig. 2). The measurements collected with the spectrometer were transformed to the space of the spectral camera by following the procedure described in the “Data processing” section. For this transformation, the transmission profile of the 35 mm fixed focal length lens, the transmission profile of the bandpass filter (FGB37, Thorlabs, Newton, United States), and the filter response of the camera (Fig. 3) were considered (Fig. 2b)), including the second order peaks, which are characteristic of some camera bands. For this purpose, the relative irradiance of the Xenon light source (IP20, Richard Wolf GmbH, Knittlingen, Germany) was measured with a spectrometer (HR2000 + , Ocean Insight, Orlando, United States) that was calibrated with a calibrated light source (HL-2000, Ocean Insight, Orlando, United States). The relative irradiance was measured by reflecting the light on a Spectralon® tile and collecting the reflected light with a cosine corrector (CCSA1, Thorlabs, Newton, United States) attached to an optical fiber.

**Fig. 4:**
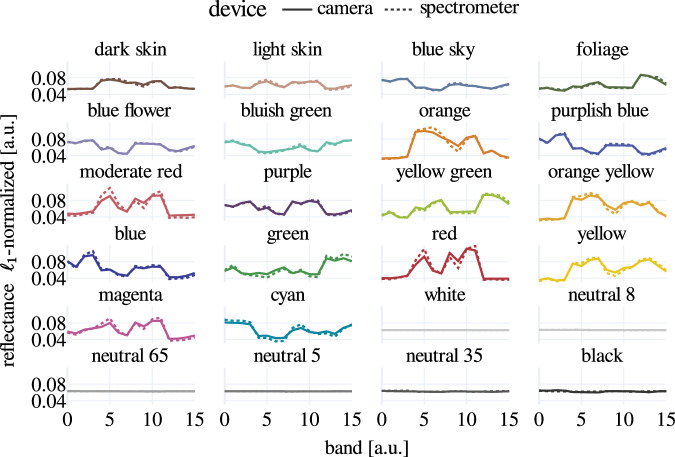
The measurements of the color checkerboard match between spectral camera and spectrometer. For comparison purposes, the data recorded with the spectral camera and the spectrometer were ${\ell }_{1}$-normalized.

The comparison of the spectral camera and transformed spectrometer measurements shows overall good agreement (Fig. 4) for most color tiles. The deviation is largest for the color tile *moderate red* (0.03 Euclidean distance) and smallest for the color tile *white* (0.0007 Euclidean distance).

**Fig. 5:**
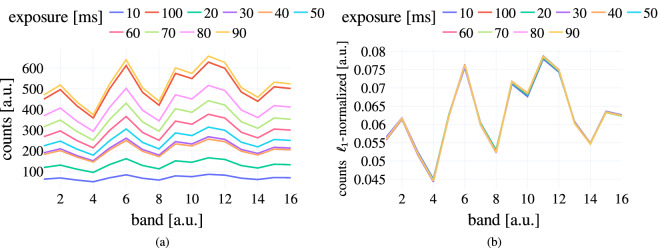
Multiplicative factors can be removed by ${\ell }_{1}$-normalization. White reference recordings at different integration times (**a**) reveal changes in spectra that can be eliminated by applying (**b**) ${\ell }_{1}$-normalization.

While the integration time of the spectral camera was consistently the same for all frames of each individual subject, we allowed it to vary between subjects to ensure the absence of undersaturated or oversaturated pixels on the palm surfaces. Although variations in the integration time alter the absolute values recorded with the spectral camera, these alterations are of a linear nature and can hence be eliminated by applying an ${\ell }_{1}$-normalization across all channels. To validate this normalization approach, we recorded spectral images of a Spectralon® tile with integration times ranging from 10 ms to 90 ms (Fig. 5a)). Figure 5b) shows that the changes introduced by different integration times can be eliminated by applying the ${\ell }_{1}$-normalization. Therefore, ${\ell }_{1}$-normalization was used in all analyses presented in this manuscript.

### Data characterization

**Fig. 6:**
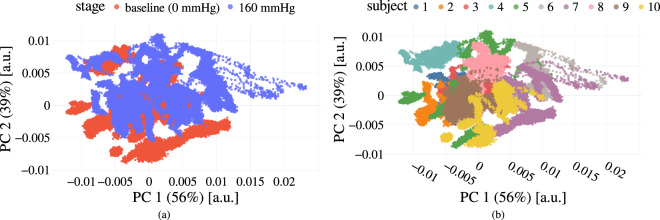
Principal Component Analysis (**PCA) reveals high inter-subject variability**. The data from different perfusion states across all subjects do not form distinctive clusters (**a**), while the data from different subjects do (**b**). The first two Principal Components (PCs) capture a large fraction of the observed variability (56% and 39%).

We analyzed the distribution of the spectral data of all subjects by performing a Principal Component Analysis (PCA) on all tracked and ${\ell }_{1}$-normalized ROIs. While the data from different functional tissue states (baseline vs. 160 mmHg) do not form distinctive clusters, the data from different subjects do (Fig. 6). This indicates that personalized approaches might be better suited for the development of new functional imaging methods.

**Fig. 7:**
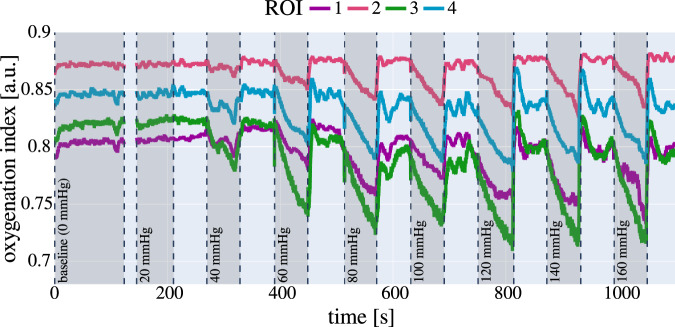
Oxygenation changes can be clearly observed for all pressure levels greater than 20 mmHg (subject 5). The oxygenation index was computed with a previously proposed machine learning-based method^11^. The gray vertical areas represent baseline measurements (perfused) or time periods where pressure was applied to the upper arm, the lighter areas between them correspond to resting states where no pressure was applied to the upper arm. The gap between the baseline and the 20 mmHg stage was used to set up and inflate the manual pressure cuff.

**Fig. 8:**
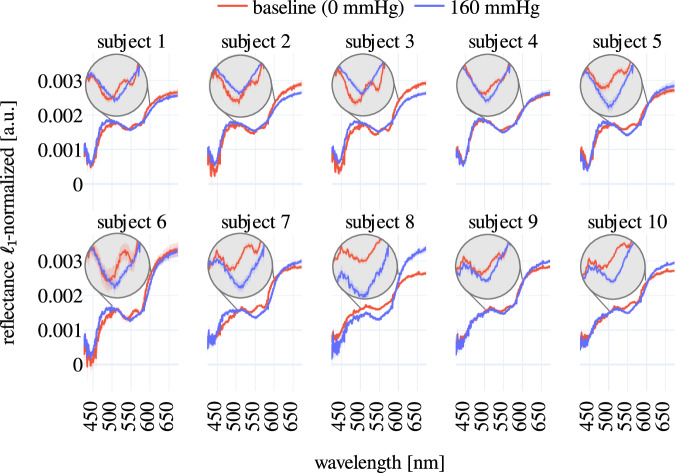
Characteristic double peak of perfused tissue (baseline) is less prominent in spectrometer measurements when applying a pressure of 160 mmHg. The solid lines correspond to the mean value within ROI 4, and the translucent bands represent the standard deviation across different ROI.

To evaluate the possibility of monitoring functional changes in tissue based on the SPECTRALPACA^27^dataset, we used an existing machine learning method based on random forest regression that was trained on synthetic data as described by the authors^11^. While changes in oxygenation were not significant for low-pressure levels (20 mmHg), they could be detected for all other pressure levels and all ROI (Fig. 7). This tendency was observed for all subjects. In a similar fashion, spectrometer measurements revealed that the double peak in reflectance measurements, that is characteristic of oxygenated blood, disappears when applying pressure to the upper arm (Fig. 8). This tendency was similarly observed for all other ROI. Both the spectral regression results and the spectrometer measurements confirm that functional changes were indeed introduced by applying pressure to the upper arms of each subject.

## Usage Notes

The SPECTRALPACA^27^ dataset is publicly available under the Creative Commons Attribute CC-BY-4.0 International License. Readers wishing to use or reference this dataset should cite this manuscript.

Even though contrast agent-free functional imaging shows particular promise in the medical field, openly accessible data in this area is particularly scarce. In contrast to other research fields, spectral data is particularly difficult to acquire and annotate. This is mainly because spectral imaging is not yet commonly used in clinical routine. As such, to the best of our knowledge, our dataset is the first of its kind: the first openly accessible dataset comprising spectral video recordings of living human tissue under different levels of perfusion. Hence, although our dataset is relatively small, it addresses an important bottleneck. In the following we indicate some, although not all, of the ways in which our dataset could be used.

### Proof-of-concept studies

New approaches aiming to develop methods for perfusion monitoring based on spectral imaging could use our dataset for first proof-of-concept studies.

### Generalization

Most of the methods currently published regarding functional imaging based on spectral imaging have been tailored to specific devices. Our dataset offers the opportunity to assess a method’s generalization capabilities.

### Comparability

Along with generalizability, the fair comparison of different methods is equally relevant. Our dataset could provide a way to compare different approaches to functional imaging on the same data, thus improving the reliability of future methodology.

### Synthetic data generation

Recently, synthetic data generation has become popular for the generation of diffuse reflectance data paired with ground truth functional properties. However, most approaches rely on simple multi-layer tissue models, thus ignoring complex spatial features. In this regard, our dataset could be used for validation or training of synthetic spectral data generation methods.

## Data Availability

The SPECTRALPACA^27^ dataset includes the Python code that facilitates loading of the spectral data. This code and its usage instructions can be found in the dataset archive in the folder named *code*.
